# Non-Cognitive Symptoms in Alzheimer’s Disease and Their Likely Impact on Patient Outcomes. A Scoping Review

**DOI:** 10.1007/s11940-025-00852-8

**Published:** 2025-10-07

**Authors:** Andrew Thaliath, Jagan A. Pillai

**Affiliations:** 1 Norton Neuroscience Institute Memory Center, Norton Medical Plaza III- Brownsboro, Suite 301, 4915 Norton Healthcare Blvd., Louisville, KY 40241, USA; 2 Lou Ruvo Center for Brain Health, Cleveland Clinic, Cleveland, OH 44195, USA; 3 Neurological Institute, Cleveland Clinic, Cleveland, OH 44195, USA; 4 Department of Neurology, Cleveland Clinic, Cleveland, OH 44195, USA; 5 Cleveland Clinic Lerner College of Medicine, Case Western Reserve University, 9500 Euclid Ave/U10, Cleveland, OH 44195, USA

**Keywords:** Non cognitive symptoms, Alzheimer’s disease, Quality of life, Management strategies, BPSD, MCI, Dementia, Quality of life, Clinical outcomes

## Abstract

**Purpose of Review:**

Increased understanding of the pathophysiology of Alzheimer’s disease (AD) has led to development of disease modifying therapies. The therapies primarily target measures of cognitive decline since AD has been thought of as a cognitive disorder. However, the non-cognitive symptoms seen in AD contribute to overall quality-of-life. This scoping review was undertaken to further our understanding of the non-cognitive features of AD.

**Recent Findings:**

The non-cognitive symptoms in AD range from changes in sensory perception, systemic changes, and neuropsychiatric manifestations. We targeted the following non-cognitive domains: vision, olfaction, GI, muscle, sleep, circadian rhythm, immune and behavioral symptoms as it relates to AD for this review. Non-cognitive features impact the ability of individuals to perform their activities of daily living, have safety implications and lead to increased caregiver burden. The review explores non-pharmacological and pharmacological measures targeted at the non-cognitive changes in AD.

**Summary:**

Non-cognitive symptoms contribute to significant disease burden in Alzheimer’s disease. It is important to screen for and provide supportive care for these symptoms to help improve clinical care. Incorporation of non-cognitive features of AD in clinical trials will help ascertain the true societal and economic impact of AD and that of potential therapeutics.

**Opinion Statement:**

Alzheimer’s disease (AD) is primarily recognized as a disorder of cognition; however, non-cognitive symptoms significantly contribute to disease burden and clinical presentation. These manifestations particularly behavioral symptoms and systemic changes beyond the central nervous system impact patients’ quality of life and increase caregiver stress. Often, such symptoms necessitate a transition from home-based care to more intensive settings, such as memory care facilities. As AD prevalence rises alongside an aging population, the shortage of dedicated memory care providers in community settings challenges access and quality of care. Improved awareness and early recognition of non-cognitive features among healthcare professionals can aid identification of modifiable systemic issues and allow timely behavioral management during clinical encounters. Treatment of these symptoms requires a multifaceted approach, incorporating pharmacological and non-pharmacological strategies. Non-pharmacological interventions, including tailored behavioral approaches and environmental modifications, can enhance quality of life for individuals with AD and reduce caregiver burden especially where specialized medical resources are limited.

## Introduction

Alzheimer’s disease (AD) is the leading cause of dementia worldwide and the prevalence of clinical AD is estimated to triple worldwide by the Year 2050 [1]. The direct health care cost related to AD is projected to exceed $1 trillion (about $3,100 per person in the US) during the same time [2]. This estimate does not accurately reflect the overall economic and societal impact considering the amount of unpaid care provided by caregivers of AD patients. The field of AD research has had extraordinary advances with development of anti-amyloid therapies for early AD, which have been shown to decrease the progression of cognitive decline [3]. Emerging AD therapeutics are primarily evaluated in clinical trials for efficacy in cognitive domains. However, patients with AD also have non-cognitive symptoms. These range from changes in sensory perception, systemic changes, and neuropsychiatric manifestations. Key non-cognitive features and their clinical significance in AD are summarized in Table 1. Currently, these are seldom screened consistently in clinician visits or tracked in therapeutic trials as potential outcomes of interest. Further research of the non-cognitive aspects of AD is imperative to ascertain their contribution to disease burden, to evaluate effectiveness of the new generation AD therapies, and to assess their true economic and societal costs in AD.

### Methods

This project was initiated given the limitations in current AD literature: different non-cognitive manifestations of AD have not been collated for a clinical audience. PubMed was queried using the key clinical domains of interest (vision, olfaction, GI, muscle, sleep, circadian rhythm, immune and behavioral symptoms) and Alzheimer’s disease. There was a special focus to incorporate publications from the last decade when the implementation of the NIA/AA 2011 guidelines for AD dementia and MCI from AD made diagnosis more consistent [4, 5]. Additional references from prior reviews that shed perspective on this subject were later incorporated into this review’s subsequently revised drafts and the final version. Key inclusion criteria included: English language publication or article with English translation available, accessible full text, article focus on human AD, not an editorial or commentary. However, this being a scoping review, individual study quality was not quantitatively assessed and case reports that provided clinically useful context were also included.

### Vision

Visual-spatial and visual-perceptual deficits due to neurodegeneration are well recognized in AD [6]. There is evidence of AD related structural changes in the eye including Aβ accumulation in the lens, decreased pupillary constriction with light and pallor of the optic nerve [7]. Retinal Aβ accumulation has also been reported prior to neurodegeneration in the retinal ganglion cell layer and visual cortex in AD [8]. AD patients tend to perform poorly in color discrimination when compared to healthy controls and have impaired motion detection [9, 10].

AD patients also have been reported to have a harder time recognizing familiar surroundings compared to controls in a study which investigated recognition ability in low luminance and contrast sensitivity to simulate real-world nighttime and foggy conditions [11]. This was thought to be from impaired visual search patterns of AD patients where they evaluate familiar scenes as if they were presented to them for the first time. These vision changes along with poor visual acuity in dimly lit conditions can contribute to limitations in the performance of activities of daily living and contribute to an increased risk of falls [12].

AD patients have been reported to have visual field deficits in the inferior temporal and nasal aspects in the arcuate regions when objectively measured using Humphrey’s automated perimeter [13].AD patients also have impaired directional motion detection that correlates with their severity of dementia [14]. They also tend to take more time and make more errors when they attempt to fixate their gaze on a stationary object, likely from difficulty in suppressing reflexive saccades [15, 16].AD patients have further been reported to have impaired depth perception (stereopsis) thought to be due to an interpretation deficit [17].

The changes with visual field deficits, motion detection and depth perception have significant safety implications especially when someone is driving. A small observational study has demonstrated that individuals with AD were 4.7 times more likely to get into a car accident when compared to age matched controls [18]. It is important to have mechanisms in place to evaluate individuals with AD to ensure safety either with Occupational Therapy or at select Department of Motor Vehicle locations. Decluttering the home environment and having high contrast surfaces to help with object recognition may help with reducing the risk of falls. Ophthalmology clinics may help with screening for AD once ocular biomarkers are better characterized in the near future [19].

### Olfaction

Olfactory changes have been reported as one of the earlier symptoms in AD [20]. Olfactory abilities have an age-dependent decline and can impact nutrition, cognitive function, and can be a prodromal symptom before onset of dementia [21]. A recent study from the National Social Life Health and Aging Project that looked at *APOE*ε4 allele association with olfaction and cognition found that there is decline of odor sensitivity (detecting them) earlier than odor identification (recognizing and naming them) or even cognition [22].

In MCI subjects, those who had deficits on olfactory testing (despite no reported subjective olfactory symptoms) were found more likely to develop AD dementia on subsequent follow-up [23]. Further evidence comes from studies using the Pocket Smell test which is a 3-item scratch and sniff test used for olfactory testing [24]. One study utilizing the Pocket Smell test demonstrated olfactory disturbance in close to 100% of AD compared to 15% of vascular dementia and none in depressed patients [25]. A recent meta-analysis demonstrated AD patients had significantly lower olfactory identification scores compared to MCI however had much higher identification scores when compared to Lewy body Dementia [26]. Olfactory dysfunction can also be seen in other neurodegenerative disorders including Parkinson’s disease, thereby limiting its use as a biomarker specifically for AD [27]. There is also neuroimaging correlate with smaller volumes of the olfactory bulbs and the primary olfactory cortex in AD compared to controls [28].

The change in olfactory perception also adversely affects the taste of food and can lead to decreased food intake and nutritional deficiencies. Studies have demonstrated that the olfactory and behavioral changes are associated with the preceding weight loss that is seen at least one to two decades prior to the formal diagnosis of dementia [29]. It is important to monitor weight changes, evaluate dietary intake, screen for nutritional deficiencies, and provide nutritional supplementation, or provide support through the help of a dietician as necessary. There are limited studies of aromatherapy, some with essential oils in individuals with dementia that have shown improvement in sleep and appetite [30].

### Gastrointestinal Tract

The enteric nervous system (ENS) controls gut motility. AD patients have reported lower constipation rates [31]. AD medications including acetylcholinesterase medications are also known to be associated with gastrointestinal symptoms such as nausea, vomiting, and diarrhea that are dose related and reversible [32, 33]. Amyloid precursor protein (APP) is expressed in enteric neurons and glia, suggesting that AD pathology might involve ENS. However, studies on this topic have yielded mixed results [34]. Gut motility changes have also been attributed to small intestinal bacterial overgrowth with symptoms of bloating, nausea, cramping, that are reported to be higher in AD [31].

Changes in oral health with aging are also a predictor of dementia risk. Periodontal disease which is known to cause tooth loss has been associated with increased brain beta amyloid load demonstrated using a PET (Positron Emission Tomography) scan [35]. Porphyromonas gingivalis an anaerobic bacteria associated with periodontitis produces gingipains a toxic protease which has been identified in brains of AD patients [36]. Proliferation of oral anaerobic bacteria in the oral microbiome has been hypothesized to create a proinflammatory response that could potentially weaken the blood brain barrier and may contribute to AD pathophysiology [37]. Changes in gut microbiome are thought to increase intestinal permeability with release of gut bacteria and metabolites into circulation which leads to proinflammatory changes in the nervous system along with cognitive outcomes related to AD [38]. Comparison of incidence rates of serious GI events, including ulceration, perforation, or bleeding in the upper or lower GI tract in individuals aged 65 and older, noted AD patients had higher incidence rates of upper and lower GI events. This association is yet to be replicated and the plausible causes unclear [39].

Practicing good oral hygiene is important to maintain good oral health that is also being investigated for its AD related outcomes. Dietary changes aimed at higher fiber, low-fat diet may help improve gut health and gut microbiota diversity. There are suggestions that probiotics could alter the gut microbiome and possibly impact AD outcomes [40].

### Skeletal Muscle

The skeletal muscle undergoes age-related changes with loss of muscle mass and strength, commonly referred to as sarcopenia [41]. Low handgrip strength has a positive correlation with cognitive decline even after controlling for other comorbidities [42]. Loss of muscle tissue has been implicated with cognitive decline related to cytokine release from skeletal muscle mediating the muscle-brain crosstalk [43]. Myokines (skeletal muscle cytokines) may exert neuro-immunomodulatory changes by reducing oxidative stress and increasing autophagy [44]. Irisin, a specific myokine released with exercise, has been shown to promote cognitive function through Brain-derived neurotrophic factor (BDNF) signaling [45]. Mitochondria are also thought to play a role in the muscle-brain axis by release of myokines and exercise induced adaptations [46].

Sarcopenia is a major contributor to frailty which makes an aging individual physically dependent on others [47]. There is association between frailty and AD biomarkers postulated to be due to either similar pro-inflammatory response causing damage or due to faulty repair mechanism that is unable to keep up with the skeletal muscle damage over time [48]. There is growing evidence for the role of aerobic exercise in cognition in AD with superior benefit in groups exercising for 30 min per session and about 150 min per week [49]. Incorporation of aerobic exercise as a lifestyle modification will help improve musculoskeletal fitness and promote physical independence. Individuals who may have physical limitations for aerobic exercise should be encouraged to work with therapy services for gait training and fall prevention.

There is an association with Inclusion Body Myositis (IBM) and AD. IBM is the most common myopathy after the age of 50, presenting progressive asymmetric weakness commonly in the quadriceps or finger flexors [50]. Muscle biopsy in IBM usually demonstrates endomysial inflammation with lymphocytes surrounding or invading a nonnecrotic myofiber along with the characteristic rimmed vacuoles [51]. There is emerging evidence of deposition of amyloid beta and phosphorylated tau in muscle fibril in individuals affected with IBM like underlying pathology in AD [52]. Supportive evidence comes from transgenic expression of human amyloid precursor protein (APP) inducing IBM like changes [53]. There are case reports of coexisting IBM and AD in individuals postulated due to similar underlying pathogenesis affecting different organ systems [54]. Evaluation of muscle mass and atrophy patterns are essential in clinical AD to characterize co-existing myopathies.

### Sleep and Circadian Rhythm

AD patients tend to have peak body temperature and peak motor activity occurring later during the day compared to normal subjects [55]. This correlates with the behavioral manifestations of AD being more prominent during later hours of the day, and it further aggravates the circadian rhythm disruption. There is also evidence of nighttime disruption in melatonin levels in AD patients when compared to age matched controls [56]. A study examining dim light melatonin onset as a surrogate for circadian rhythm found that earlier circadian timing and reduced levels of melatonin was found to be associated with reduced hippocampal volumes [57].

Melatonin is widely used for circadian rhythm related disorders and may be useful as an adjunct to help normalize the disruption. A meta-analysis demonstrated that low dose (2–2.9 mg) and medium term (6 months to 1 year) supplementation with melatonin may decrease cognitive decline as measured by Mini Mental Status Exam (MMSE) score [58]. Other strategies include adequate exposure to light during the daytime through outdoor activities or in home supplemental lighting, may help with behavioral symptoms noted in AD related to sun downing [59].

In AD there is relative increase of Non-Rapid Eye Movement (NREM) stage 1 and 2 sleep with relative decrease of slow wave sleep (SWS) in early AD and decreased duration of Rapid Eye Movement (REM) sleep in late-stage AD [60]. The relative decrease of NREM SWS in humans is associated with increased Aβ deposition in the mesial prefrontal cortex and also impairment of hippocampal episodic memory consolidation [61]. AD patients tend to have sleep fragmentation with either an increase in the amount of awake time or number of awakenings during the night compared to controls [62]. AD patients also tend to have a higher oxygen desaturation index (ODI) and higher apnea hypopnea index (AHI) compared to controls [63]. Obstructive sleep apnea is associated with increased the risk of cognitive decline and if treated may help contribute to mitigation of cognitive decline in the context of AD [64].

Screening for sleep disorders using sleep questionnaires should become a routine part of clinical care. This should be supplemented with a sleep study when indicated since there are potential treatment options if sleep disordered breathing is identified. Other interventions aimed at improving overall sleep quality using both nonpharmacological measures including optimizing sleep hygiene and medications to help with sleep initiation should be considered. Meta-analyses, have reported only small effects of bright light therapy in AD dementia [65]. Mixed results were also reported on a randomized control clinical trial, however the authors concluded that there was still reason to assume that BLT may be beneficial for people with severe dementia [66].

### Behavioral and Psychological Symptoms of Dementia

Behavioral and psychological symptoms of dementia (BPSD) are a group of neuropsychiatric symptoms that may affect up to 90% of patients with dementia and can vary from agitation, disinhibition, irritability, depression, anxiety, apathy, hallucinations, and or delusions [67]. BPSD of depression, anxiety and apathy are thought to be more prevalent and might present before cognitive changes in AD patients [68]. The symptoms of agitation and sleep disturbances were found to negatively impact a patient’s quality of life and increase caregiver burden [69]. AD patients with BPSD have poorer clinical outcomes with increased mortality and readmission rates in cases of hospital admission [70]. The clinical symptomatology and neuroimaging correlates may be helpful to provide prognosis on expected BPSD and this knowledge can be generalized to variants of AD [71]. Temporoparietal involvement in behavioral/dysexecutive variant AD tend to have more overall BPSD compared to other variants of AD [72].The parietal occipital involvement with visuospatial involvement in Posterior Cortical Atrophy(PCA) tend to have more visual hallucinations and REM Sleep behavior changes whereas, left temporal parietal involvement with language changes in logogenic Primary progressive aphasia (PPA) may have more personality changes [73].

Clinic visits should incorporate active management of BPSD to help improve the patient’s quality of life and decrease caregiver burden. Management of BPSD should incorporate both non pharmaceutical measures and pharmacological interventions. Insufficient light exposure is associated with increased BPSD and sundowning in nursing home residents [74]. Ambient bright light has shown effects in reducing depression and agitation symptoms related to BPSD [75]. Increasing daylight exposure and having structured scheduled activities during the daytime may help ameliorate some of the BPSD. Other non-pharmacological measures including music therapy, therapeutic touch, and aromatherapy might be useful in BPSD management [76]. Cognitive interventional therapy, which is aimed at improving an individual’s cognitive and functional capacity, may also contribute to improving BPSD [77].

Medications approved for use in AD like acetylcholinesterase inhibitors and memantine may help with negative and positive neuropsychiatric symptoms, respectively [78]. Off label use of antidepressants, antipsychotics, mood stabilizers and stimulants to target various neuropsychiatric manifestation of AD is also common in clinical practice [79]. Brexiprazole is a recently approved specifically for agitation associated with Alzheimer’s. Brexiprazole showed significant improvement in agitation when compared to placebo in a 12-week double blind study [80].

### Immune System

Considerable body of literature suggests significant immune changes in both the peripheral and central nervous systems in AD. There is a concomitant change in the integrity of the blood brain barrier in this context [81]. Increased levels of anti- and pro-inflammatory cytokines are found both in the serum and cerebrospinal fluid (CSF) in AD [82]. Neutrophil-to-lymphocyte (NLR) which is a marker for systemic inflammation is elevated in AD patients compared to healthy controls contributing to the inflammatory cascade [83]. A population-based cohort study of innate immunity (granulocytes and platelets) and adaptive immunity (lymphocytes) found a shift towards innate immunity had greater risk of dementia [84]. A decrease in memory T cells and increase in terminally differentiated effector memory T _EMRA_cells is linked to decreased cognition as seen in AD [85]. The exaggerated proinflammatory response during systemic illness or delirium also is thought to contribute to prolonging effects from these conditions. Proinflammatory cytokines changes in the plasma and CSF may impact AD clinical outcomes [86, 87]. CSF C-C motif ligand 2 (CCL2) levels have been linked to more rapid clinical decline in the MCI stage of AD [88]. High peripheral inflammation levels have also been reported be associated with a sex-specific memory vulnerability relevant for AD [89].

The dysregulated immune responses in AD emphasizes the need for infection prevention strategies to prevent hospitalization and the study for role for vaccines and their efficacy in this population. It is important to minimize hospitalizations when feasible and have delirium prevention strategies in place. Exercise has neuro-immunomodulatory effects and has been demonstrated to promote hippocampal neurogenesis in animal models [90]. There are early phase clinical trials that are looking into the feasibility of pharmaceutical agents targeting microglia for slowing down cognitive decline [91].

## Conclusion

Alzheimer’s disease is considered a disorder of cognition and the non-cognitive symptoms are sometimes overlooked. The non-cognitive symptoms described in this review contribute to poorer quality of life in AD and increased caregiver burden. However, these symptoms are not often screened for during routine clinical visits and sometimes remain unaddressed. There are supportive therapies available to manage these symptoms as outlined in Table 2. Screening for them early in the disease process can help maintain a better quality of life. We suggest incorporating these strategies into clinical practice to improve patient and caregiver outcomes in AD. There is enough evidence to consider including most of these non-cognitive domains among the secondary outcome measures in future clinical trials when exploring the role for novel therapeutics in AD. Future studies should include these non-cognitive symptoms to better our understanding of the impact it has on AD patients.

## Figures and Tables

**Table 1:** Key non-cognitive manifestations and their clinical significance in AD

Non-cognitive manifestation	Clinical significance
Vision	Difficulties with performing Activities of Daily Living (ADL), Instrumental Activities of Daily Living (IADL), reading, and other leisure activities
Olfaction	Effect on taste and decreased oral intake which may lead to weight loss and nutritional deficiencies
Gastrointestinal	Oral and gut microbiome changes which may contribute to pathophysiology
Skeletal Muscle	Frailty and Sarcopenia may lead to being more dependent on others
Circadian Rhythm	Sun downing
Sleep changes	Disrupted sleep-wake cycle
Behavioral and psychological symptoms of Dementia	Effect on caregiver burden and quality of life
Immune system	Exaggerated responses to infections or delirium

**Table 2:** Strategies to help improve patient quality of life (QOL) in key non-cognitive manifestations of AD

Non-Cognitive Manifestation	Strategies to improve QOL
Vision	Decluttering home environment Optimize lighting Driving Evaluation
Olfaction	Screen for weight loss due to effect on appetite Track weight changes over time, especially in populations at higher risk. Evaluate dietary intake Implement nutritional support and address underlying causes.
Gastrointestinal	Oral hygiene Dietary changes
Skeletal Muscle	Aerobic Exercise Physical Therapy
Circadian Rhythm	Sleep hygiene Medications -Melatonin Ambient Bright Light Exposure
Sleep	Sleep questionnaire and Sleep Study Treat sleep-disordered breathing
Behavioral and psychological symptoms of Dementia	Nonpharmacological measures Pharmaceutical measures
Immune system	Infection and Delirium prevention measures

## Data Availability

No datasets were generated or analysed during the current study.
